# Supplementary material for: Tuning antibody stability and function by rational designs of framework mutations
Source: MAbs. 2025 Jul 13;17(1):2532117. doi: 10.1080/19420862.2025.2532117 (PMC12269682; doi:10.1080/19420862.2025.2532117)
Supplement: Supplementary_Materials.docx [file KMAB_A_2532117_SM1408.docx]

**Supplementary Materials**

**Supplementary Tables**

Supplementary Table S1. Rosetta saturated mutagenesis results for the VH and VL domains. (XLSX)

Supplementary Table S2. Probability scores for wild-type (WT) and respective mutations (MUT) returned by AntiBERTy, AbLang2, AntiBERTa2 and ESM-2, for all experimentally characterized mutations considered in this analysis.

|  |  | AntiBERTy | |  | AbLang2 | |  | AntiBERTa2 | |  | ESM-2 | |
| --- | --- | --- | --- | --- | --- | --- | --- | --- | --- | --- | --- | --- |
|  |  | WT score | MUT score |  | WT score | MUT score |  | WT score | MUT score |  | WT score | MUT score |
| VH | A40R | 0.903 | 0.001 |  | 0.403 | 0.006 |  | 0.726 | 0.008 |  | 0.252 | 0.053 |
|  | K43D | 0.918 | 1.60E-04 |  | 0.618 | 0.001 |  | 0.807 | 4.33E-04 |  | 0.637 | 9.52E-05 |
|  | R50S | 0.007 | 0.024 |  | 0.006 | 0.003 |  | 0.040 | 0.101 |  | 0.082 | 0.157 |
|  | R59N | 0.010 | 0.100 |  | 0.017 | 0.071 |  | 0.031 | 0.116 |  | 0.011 | 0.021 |
|  | S85N | 0.936 | 0.018 |  | 0.617 | 0.120 |  | 0.801 | 0.052 |  | 0.632 | 0.101 |
|  | R87T | 0.923 | 0.020 |  | 0.635 | 0.118 |  | 0.544 | 0.086 |  | 0.188 | 0.137 |
| VL | Q89A | 0.941 | 1.38E-04 |  | 0.646 | 3.17E-04 |  | 0.044 | 0.588 |  | 0.719 | 0.052 |
|  | Q89H | 0.941 | 0.008 |  | 0.646 | 0.138 |  | 0.044 | 0.002 |  | 0.719 | 0.031 |

Supplementary Table S3. Summary of molecular dynamics simulations.

| **System** | **Number of replicas** | **Simulation time** |
| --- | --- | --- |
| WT with HER2 bound | 3 | 1 μs |
| WT without HER2 (i.e. unbound) | 3 | 1 μs |
| VH R50S+R59N with HER2 | 4 | 1 μs (2 replicas)  0.5 μs (2 replicas) |
| VH S85N+R87T with HER2 | 3 | 1 μs |
| VL Q89A with HER2 | 3 | 1 μs |
| VL Q89H with HER2 | 3 | 1 μs |

Supplementary Table S4. Criteria used for accessing residue interactions in MD trajectories. COM, center of mass.

| **Interaction Type** | **Distance cutoff (nm)** | **Angle cutoff (degrees)** | **Notes** | **Reference** |
| --- | --- | --- | --- | --- |
| π–π stacking | ≤ 0.5  (COM–COM) | ≤ 90  (norm-norm) | Norm defined on the aromatic ring. | [1][2] |
| XH-ring (X = C, N) | ≤ 0.4  (X–COM_ring_) | > 45  (X-H-COM_ring_) |  | [3][4] |

**Supplementary Figures**


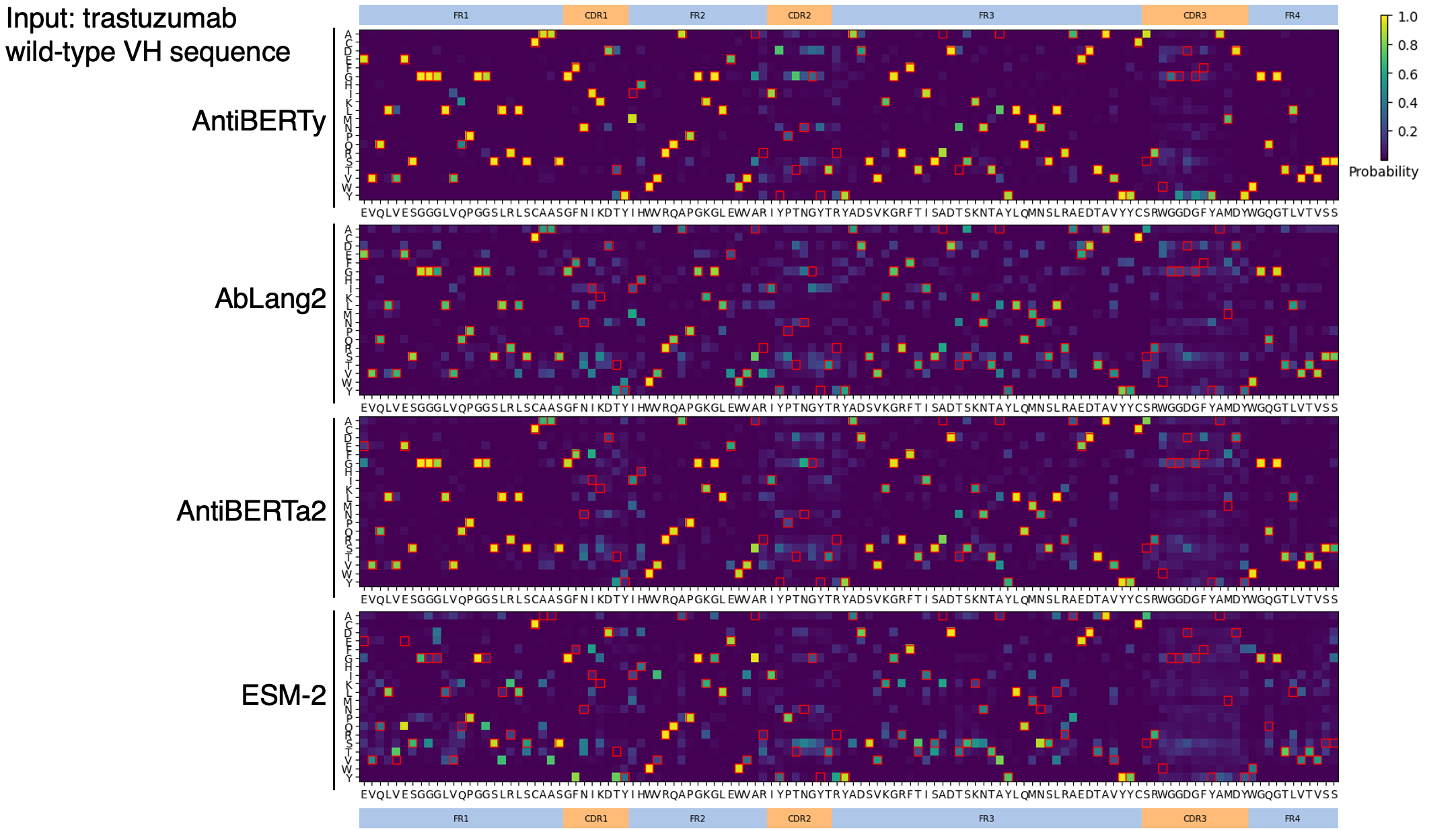


**Supplementary Figure S1.** Per-position probability returned by AntiBERTy, AbLang2, AntiBERTa2 and ESM-2 models using the wild-type trastuzumab VH sequence as input. Each column in the heatmaps corresponds to individual positions in the trastuzumab VH domain, and each row corresponds to the mutant amino acid. The wild-type residues for each position are highlighted with squares with red outlines.


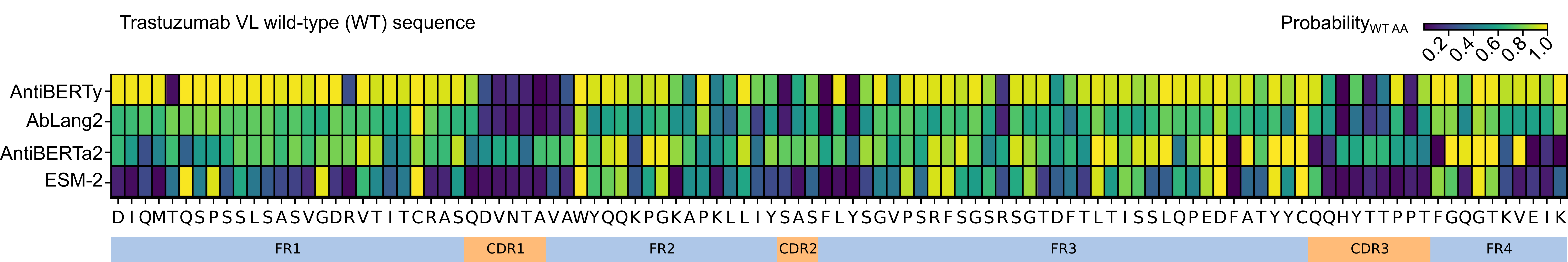


**Supplementary Figure S2**. Heatmap illustrating position-specific probability for the wild-type amino acid (columns) along the trastuzumab VL sequence using different language models (rows). FW and CDR regions are delimited below the heatmap.


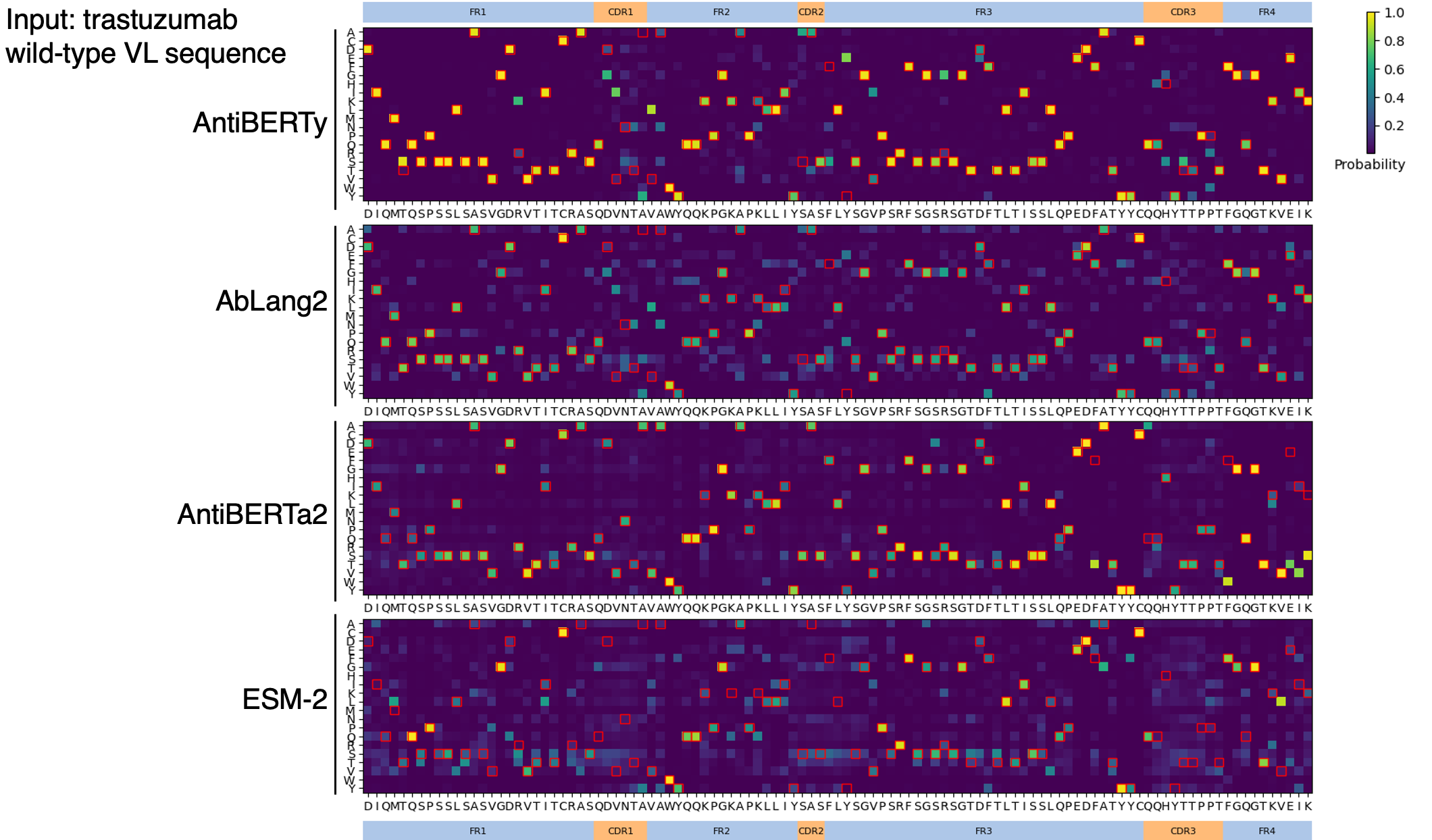


**Supplementary Figure S3.** Per-position probability returned by AntiBERTy, AbLang2, AntiBERTa2 and ESM-2 models using the wild-type trastuzumab VL sequence as input. Each column in the heatmaps corresponds to individual positions in the trastuzumab VH domain, and each row corresponds to the mutant amino acid. The wild-type residues for each position are highlighted with squares with red outlines.


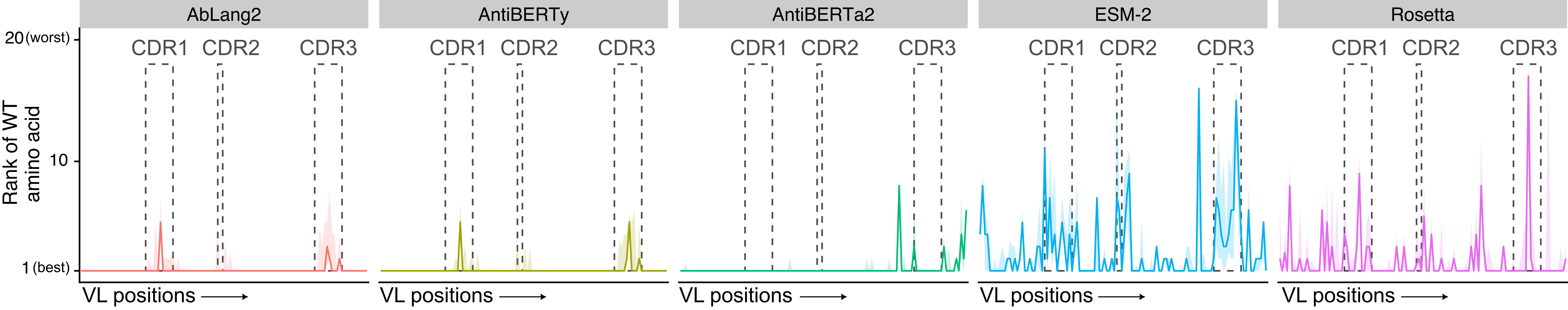


**Supplementary Figure S4**. Rank of wild-type amino acid (vertical axis, 1 = best, 20 = worst) along the VL sequences (horizontal axis) of n = 55 human therapeutic antibodies using the computational approaches evaluated in this work.


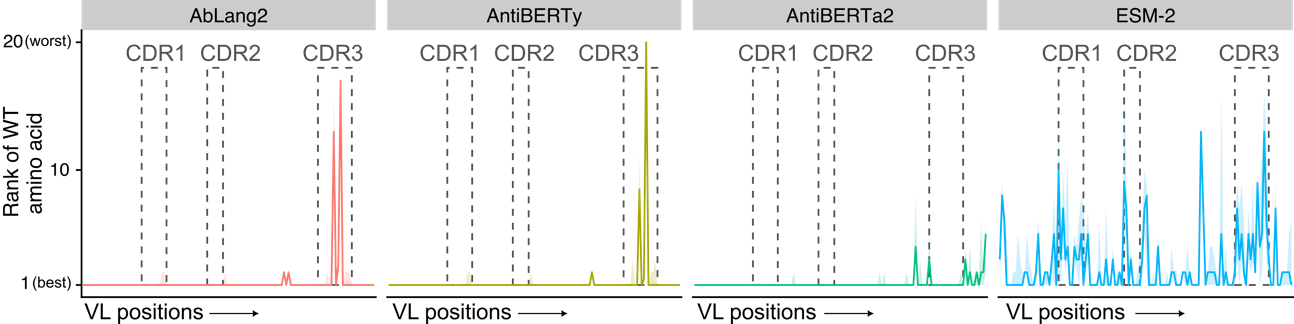


**Supplementary Figure S5**. Rank of wild-type amino acid (vertical axis, 1 = best, 20 = worst) along the VL sequences (horizontal axis) of n = 1,988 paired H-L chains from class-switched memory B cells taken from the Jaffe et al. [5] dataset.


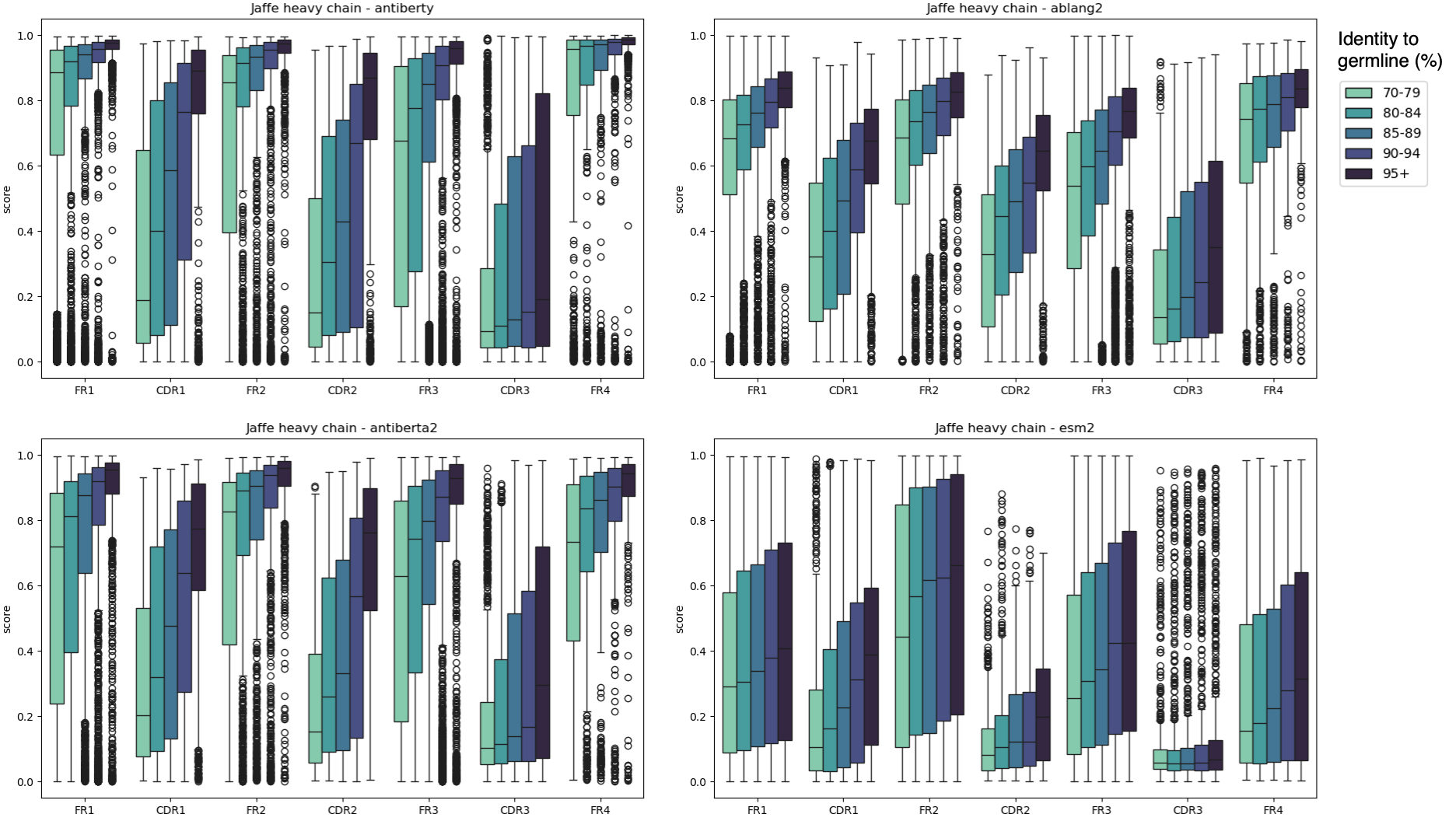


**Supplementary Figure S6**. Probability of WT residues per position returned by AntiBERTy, AbLang2, AntiBERTa2 and ESM-2 models, using VH sequences from class-switched memory B cells from Jaffe et al. (i.e. identical dataset as main text Fig. 1e) as input. The sequences were grouped by their identity to germline alleles. Residue positions were grouped by FW and CDR regions (*x*-axis).

**
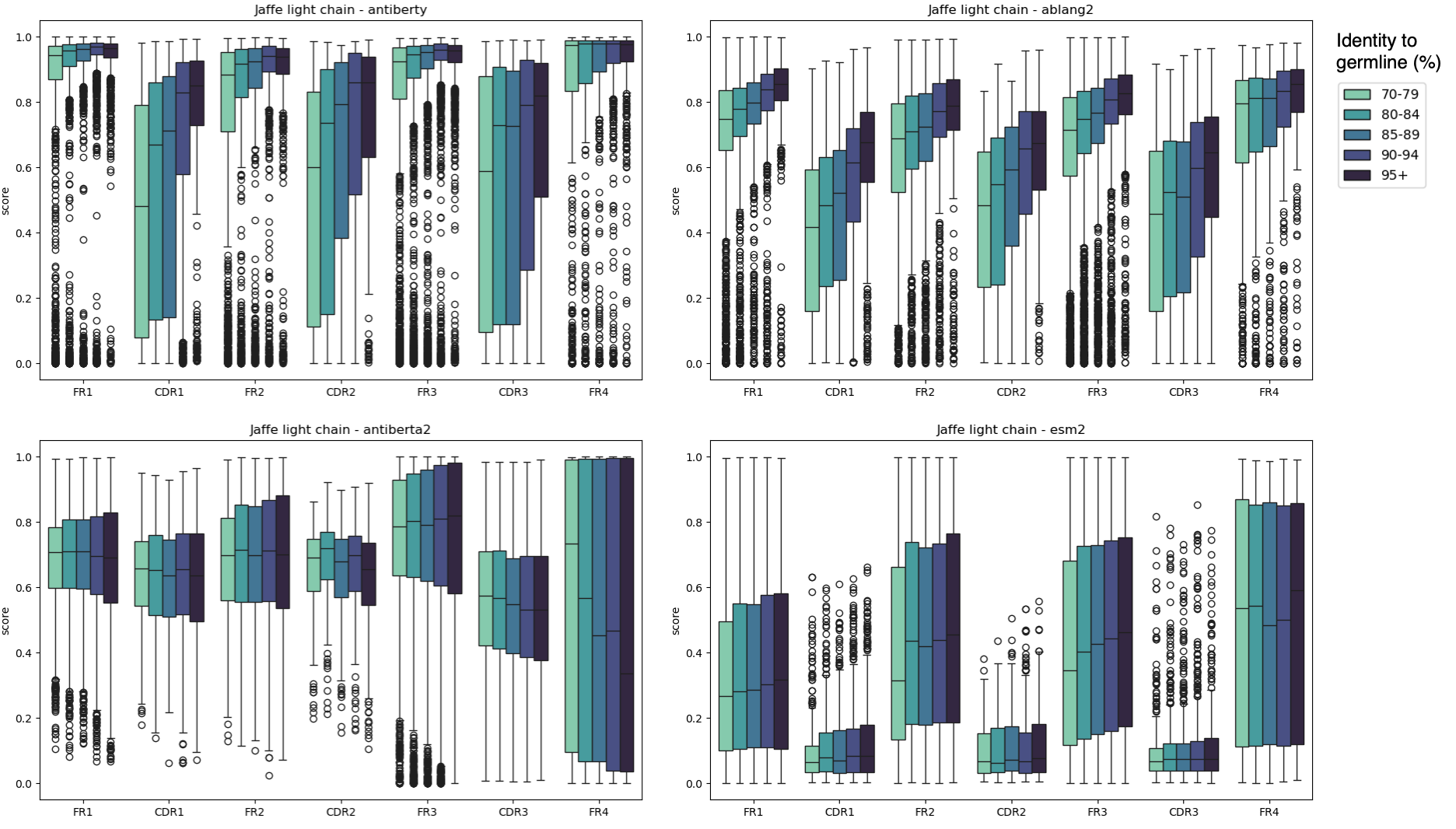
**

**Supplementary Figure S7**. Probability of WT residues per position returned by AntiBERTy, AbLang2, AntiBERTa2 and ESM-2 models, using VL sequences from class-switched memory B cells from Jaffe et al. (i.e. identical dataset as main text Fig. 1e) as input. The sequences were grouped by their identity to germline alleles. Residue positions were grouped by FW and CDR regions (*x*-axis).


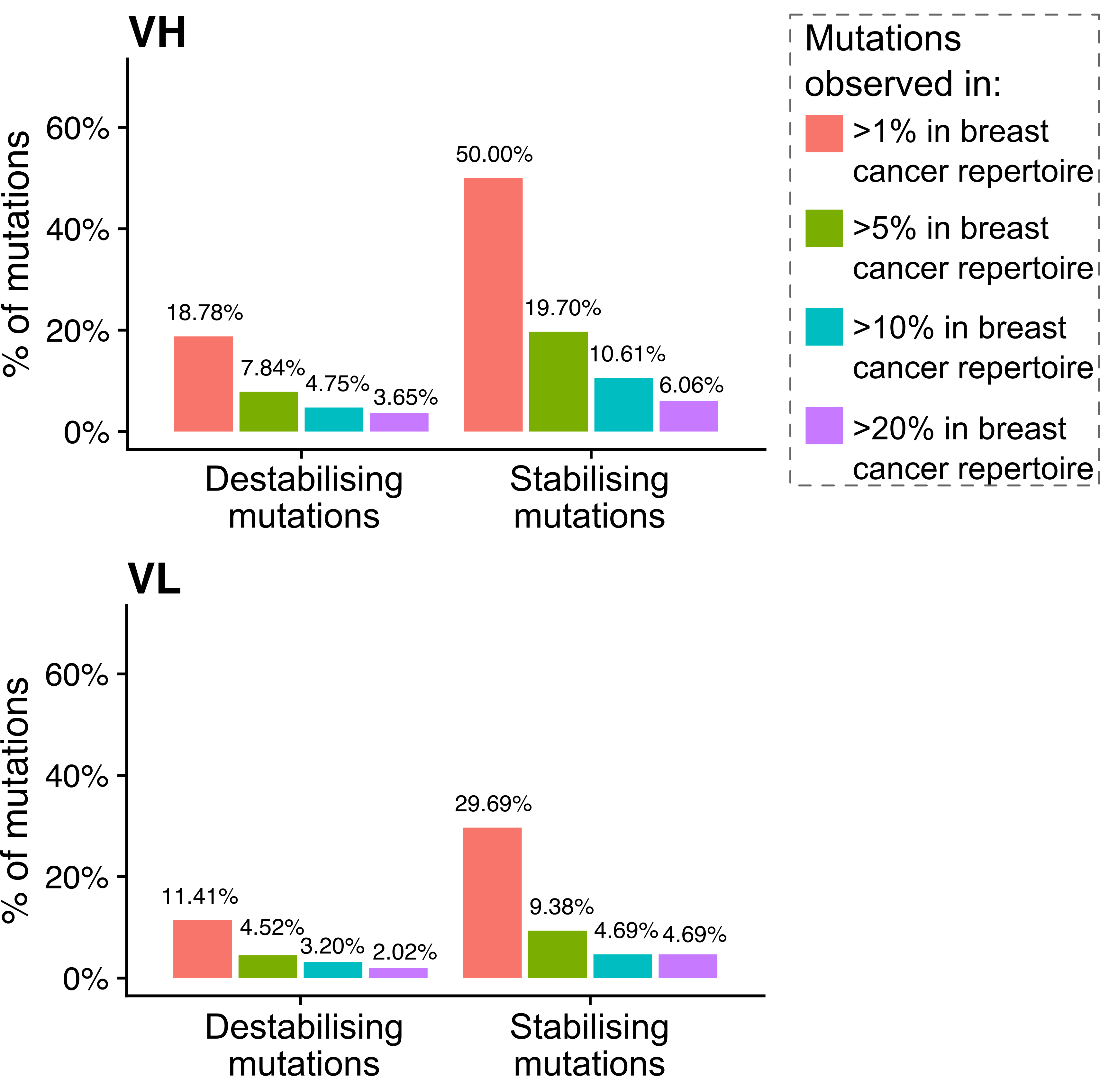


**Supplementary Figure S8**. Comparison of mutation occurrence in a breast cancer antibody repertoire dataset (Harris et al. [6]) against stabilizing mutational effect prediction from Rosetta generated in this study. Mutations were classified into destabilizing (Rosetta predicted ∆∆*G* > 0) and stabilizing (∆∆*G* < 0), and by their observed frequencies (in terms of percentage of sequences in the repertoire data).


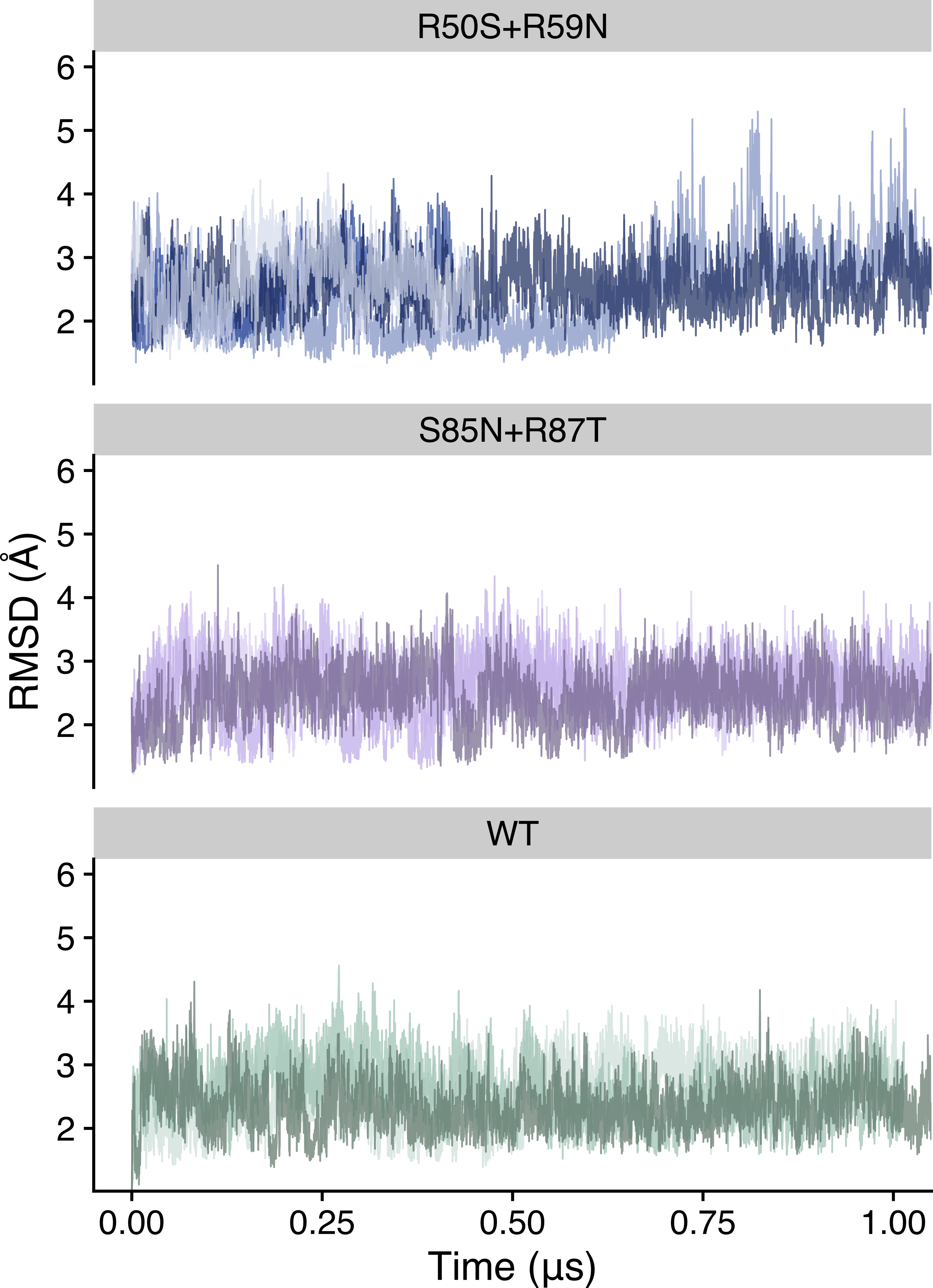


**Supplementary Figure S9**. Root-mean-squared deviation (RMSD) of MD frames across simulation time, for the WT, VH R50S+R59N and VH S85N+R87T systems. Each colour represents a replica.


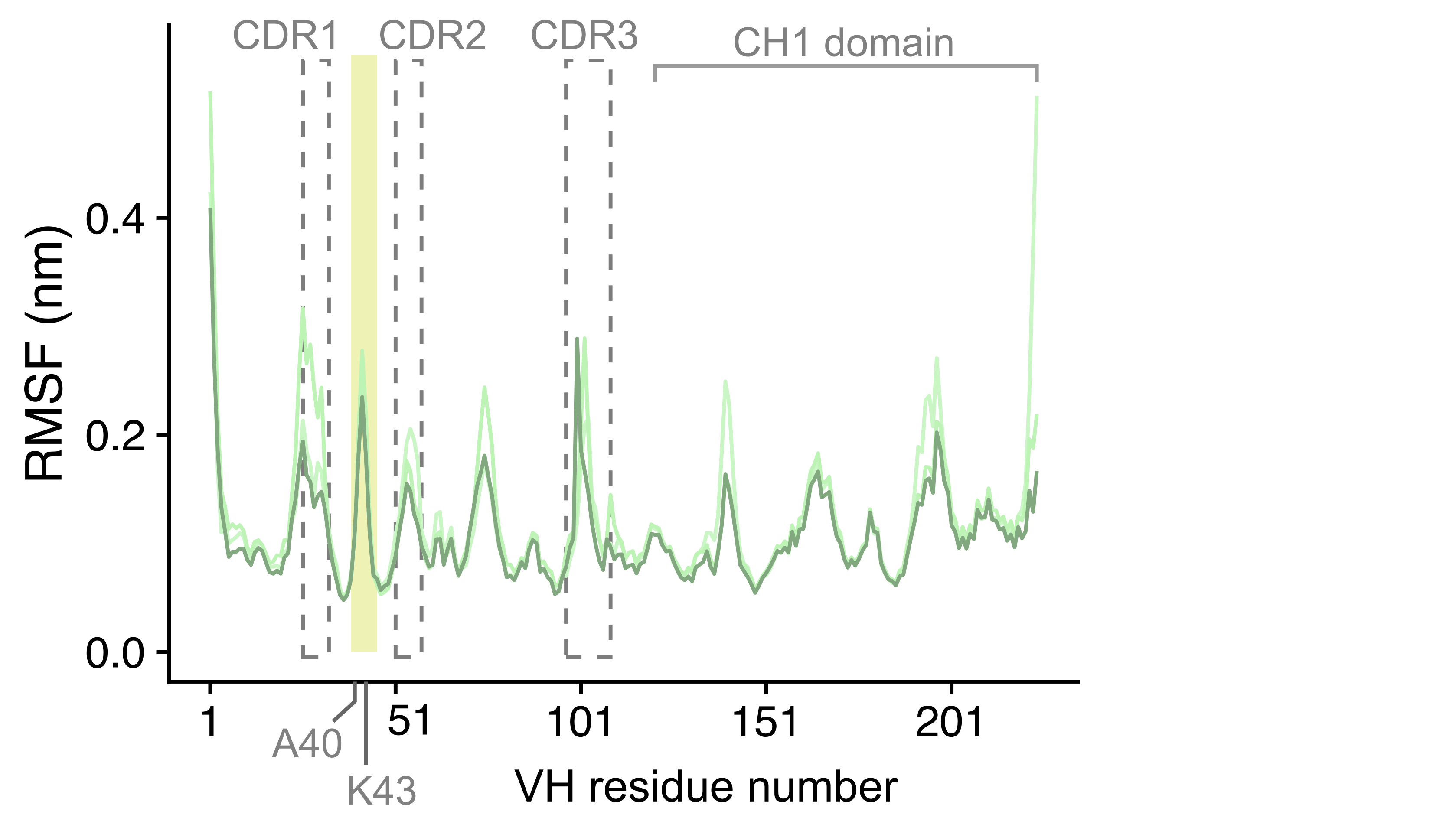


**Supplementary Figure S10**. Root-mean-squared fluctuation (RMSF) of each amino acid Cα in the WT simulations., Each color represents a replica. The regions corresponding to CDR loops and the CH1 domains are labelled in grey. The loop containing A40 and K43 is highlighted in yellow.


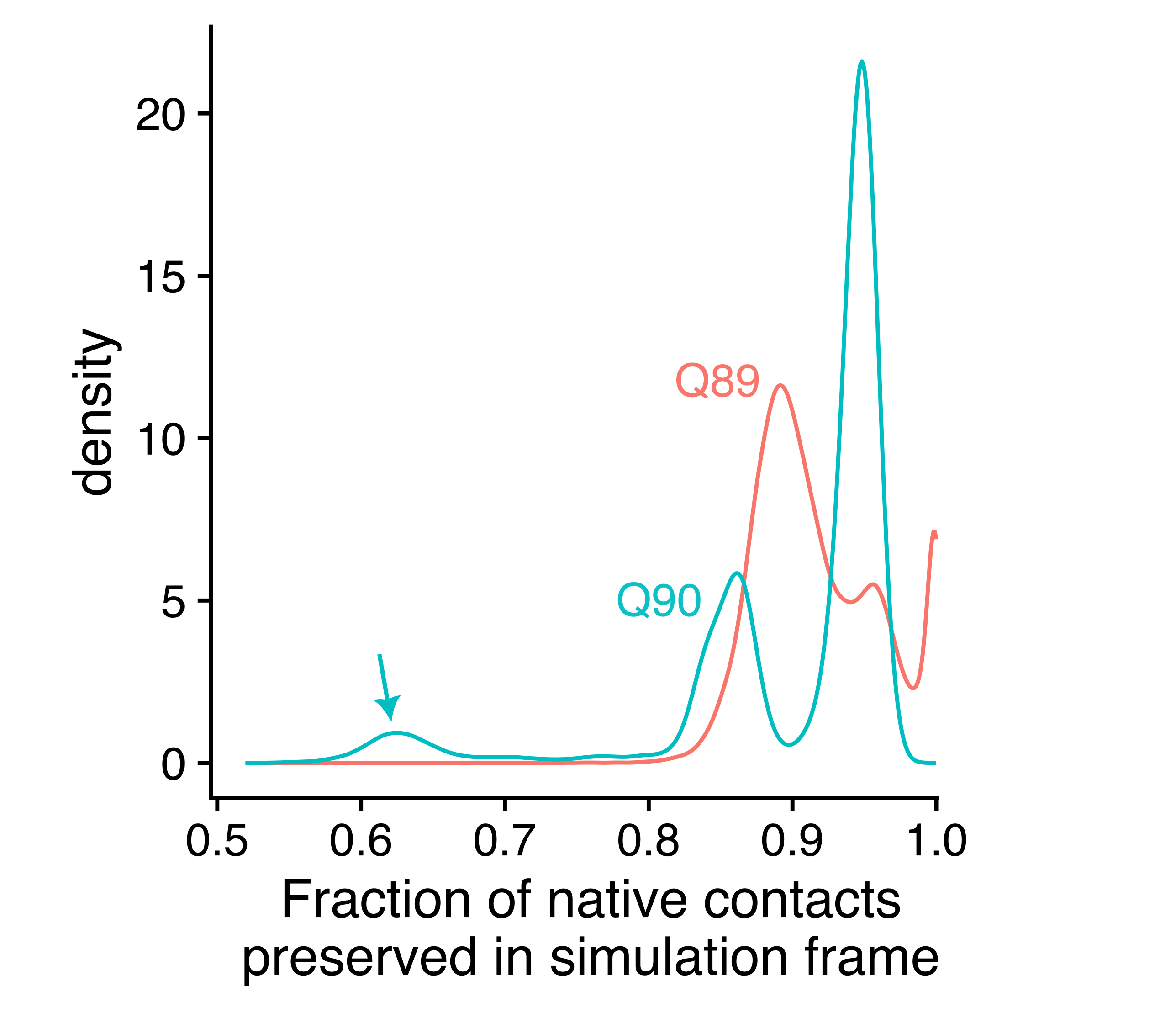


**Supplementary Figure S11**. Fraction of native contacts observed made by Q89 and Q90 in each MD simulation frame in the WT system. ‘Native’ contacts refer to contacts made by the side-chains of Q89 and Q90, observed in the starting frame and within a shell of 5Å. The subset of frames where Q90 loses native contact was highlighted with an arrow in the plot.


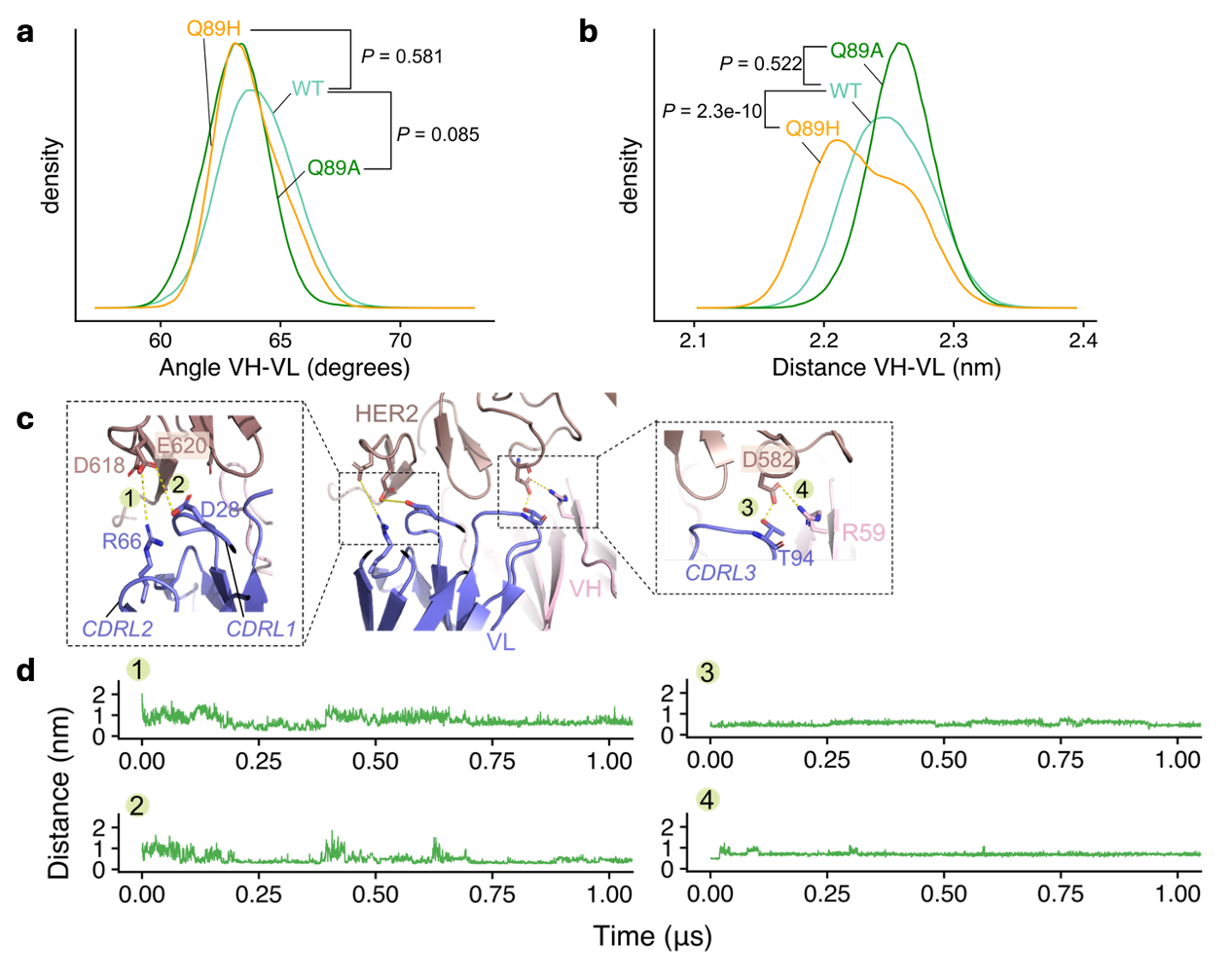


**Supplementary Figure S12**. Intermolecular interactions in trastuzumab VL Q89A. (a-b) Distribution of (a) VH-VL packing angle (calculated as in main text Figure 3d) and (b) VH-VL distance (calculated as in main text Figure 3e) in the WT, Q89A and Q89H MD trajectories. Statistical comparisons were performed with a mixed effect models using the MD replicas as random effects. (c) Structural analysis of the interface between HER2 and the VH and VL domains of trastuzumab, highlighting antigen-antibody contact for distance evaluation in (d). (d) Change in the distance between specified residue pairs in (c), in the Q89A MD trajectories. Data from one representative replica are shown.

**References**

1. Calinsky, R. & Levy, Y. Aromatic Residues in Proteins: Re-Evaluating the Geometry and Energetics of –, Cation, and CH Interactions. *J Phys Chem B* **128**, 8687–8700 (2024).
2. Calinsky, R. & Levy, Y. Histidine in Proteins: pH-Dependent Interplay between –, Cation–, and CH– Interactions. *J Chem Theory Comput* **20**, 6930–6945 (2024).
3. Wang, J. & Yao, L. Dissecting CH and NH Interactions in Two Proteins Using a Com- bined Experimental and Computational Approach. *Sci Rep* **9**, 20149 (2019).
4. Brandl, M. *et al.* C-H-interactions in proteins*. J Mol Biol* **307**, 357–377 (2001).
5. Jaffe, D. B. *et al.* Functional antibodies exhibit light chain coherence. *Nature* **611**, 352–357 (2022).
6. Harris, R. J. *et al.* Tumor-Infiltrating B Lymphocyte Profiling Identifies IgG-Biased, Clonally Expanded Prognostic Phenotypes in Triple-Negative Breast Cancer. *Cancer Res* **81**, 4290–4304 (2021).
